# Effect of lorcaserin on weight reduction in persons with obstructive sleep apnea (OSA): a combined subgroup analysis from three randomized, controlled clinical trials

**DOI:** 10.1002/osp4.340

**Published:** 2019-04-24

**Authors:** K. Fujioka, C. Perdomo, M. Malhotra

**Affiliations:** ^1^ Department of Diabetes and Endocrinology Scripps Clinic San Diego CA USA; ^2^ Neurology Business Group Eisai Inc. Woodcliff Lake NJ USA

**Keywords:** Lorcaserin, obstructive sleep apnea, sleep‐related breathing disorder, weight loss

## Abstract

**Study objectives:**

To evaluate weight loss with lorcaserin in persons with obstructive sleep apnea (OSA).

**Methods:**

This retrospective analysis evaluated weight loss of lorcaserin (10 mg twice daily) versus placebo in persons with obesity or overweight persons with OSA from a pooled database of three randomized, controlled trials. Primary end points were reductions in the baseline body weight of ≥5% and ≥10% at year 1 and overall weight change at year 1. Changes in heart rate and blood pressure were also evaluated.

**Results:**

A total of 336 persons with OSA were identified in the overall pooled population (*N* = 6,636). At year 1, more patients receiving lorcaserin lost ≥5% (47.2% lorcaserin vs. 25.6% placebo; *p* < 0.0001) and 10% (22.2% lorcaserin vs. 13.1% placebo; *p* < 0.0354) of their baseline body weight. Weight loss at year 1 was 6.4 kg versus 3.5 kg in the lorcaserin and placebo groups, respectively (*p* < 0.0001). Similar results were observed for change in blood pressure and heart rate, with responders having larger benefits. Weight loss was similar between persons with and without OSA.

**Conclusions:**

In this retrospective analysis, persons with OSA showed significant and meaningful weight loss, blood pressure and heart rate reductions in patients treated with lorcaserin versus placebo. Persons with OSA lost just as much weight as those without OSA. Health care providers can expect persons with OSA to lose weight by diet, exercise and the weight loss medication lorcaserin comparable with persons without OSA. Further prospective research is warranted to evaluate impact of weight loss on OSA and overall outcomes for patients.

## Introduction

Obstructive sleep apnea (OSA), a complete or partial halt in airflow during sleeping, results in fragmented quality of sleep that often produces an excessive level of daytime sleepiness and is associated with decreased quality of life and cognitive function, cardiovascular (CV) complications and even death in some cases 1, 2. The prevalence of OSA is increasing, afflicting at least 25 million adults in the United States. OSA occurs more frequently in men than women and is most common in older adults, with obesity 3, 4. Undertreatment is a major issue, as only 10–20% of OSA is diagnosed 5.

Obesity is a major risk factor for OSA and is modifiable, unlike many other related factors 1, 2, 6. OSA, in turn, can worsen obesity, creating a negative cyclical feedback loop 1, 7. For example, obesity can result in OSA via several well‐documented mechanisms, whereas more recent evidence suggests that OSA can worsen obesity 1. Weight loss for all persons with obesity or overweight is recommended, with studies showing that losing weight can reduce the severity of apnea and related symptoms 8, 9, 10, 11, 12. Furthermore, improvements in apnea severity are greater with more weight loss 12, 13. And even short‐term weight loss can have long‐term benefit for OSA 11. For example, in a randomized controlled study in 63 adult male persons with obesity or with moderate to severe sleep apnea, an average weight loss of 20 kg over 9 weeks was associated with a 58% improvement in sleep apnea symptoms and decline of 21 OSA events per hour 14. Based on current evidence, achieving significant and sustained weight loss should be a useful therapeutic tool in the management of OSA 1, 2.

Persons with OSA are at high risk of CV events as well as an increased risk of many additional health issues, including cognitive impairment, motor vehicle crashes, atrial fibrillation, stroke and overall mortality 15. Multiple treatments for OSA have shown reductions in apnea–hypopnea index, Epworth Sleepiness Scale scores and blood pressure 15, 16. These treatments include weight loss programmes, continuous positive airway pressure (CPAP), surgery, mandibular advancement devices as well as behavioural changes (i.e. diet and exercise).

An expert multidisciplinary panel recently convened to assess the evidence regarding the impact of various weight loss strategies on key clinical outcomes important to OSA patients noted that weight loss does improve OSA severity and obesity is a chronic disease that is a consistent contributor to OSA 17, 18. The panel recommended pharmacotherapy and/or surgical options for persons with obesity and OSA who are unsuccessful in achieving a weight loss goal through comprehensive lifestyle programmes.

Weight loss and non‐invasive CPAP are recommended for all patients, but there are no approved pharmacological therapies specifically for OSA. CPAP is considered the gold standard treatment for OSA 19. CPAP is not curative and there can be difficulties with compliance to therapy 20. Recent studies with CPAP have shown modest sleep‐related quality‐of‐life improvements but have not demonstrated a substantial impact on objective outcomes, such as CV events 15, 20, 21. In a recent post hoc analysis of the Sleep Apnea Cardiovascular Endpoints study in over 2,400 patients, long‐term CPAP use in persons with co‐morbid OSA and CV disease did not result in clinically significant weight change 22. In contrast, weight loss programmes have been shown to reduce the severity of apnea and related symptoms 8, 9.

Lorcaserin HCl is a selective serotonin 2C receptor agonist approved for weight management in persons with a body mass index (BMI) ≥30 kg m^−2^, or ≥27 kg m^−2^ with ≥1 weight‐related co‐morbidity, as an adjunct to a reduced calorie diet and increased physical activity 23. Lorcaserin, a non‐sympathomimetic, works by selectively activating the serotonin 2C receptors in the hypothalamus, which modulates food intake by triggering the proopiomelanocortin system of neurons that induces hypophagia 24. The efficacy and safety of lorcaserin were established in three phase III clinical trials in adult persons with overweight or obesity 25, 26, 27. Lorcaserin 10 mg twice daily (BID) achieved significantly more weight loss relative to diet and exercise alone after 1 year of therapy. Importantly, the use of this agent is non‐stimulating and not associated with insomnia in clinical trials.

Given the potential of OSA to potentially encourage obesity and potentially impede weight loss, the objective of this retrospective, subgroup analysis was to evaluate the effect of lorcaserin on weight loss in persons with OSA from a pooled database of three randomized, controlled phase 3 studies and to evaluate if the persons with OSA can lose weight just as successfully as persons without OSA. The main hypothesis for this analysis was that persons with OSA would attain just as much meaningful weight loss as well as blood pressure and heart rate reduction with lorcaserin as those that do not have OSA. The studies were conducted at academic and private research sites in the United States under the guidelines of the Declaration of Helsinki. Institutional review boards reviewed and approved the protocols for each research site. All patients provided written informed consent before participation in the trials.

## Patients and methods

### Trial design

The detailed design of the three trials included in this analysis, the study‐specific efficacy and safety end points, study procedures and the inclusion and exclusion criteria have been published previously 25, 26, 27. Briefly, BLOOM (NCT00395135, ClinicalTrials.gov), BLOSSOM (NCT00603902, ClinicalTrials.gov) and BLOOM‐DM (NCT00603291, ClinicalTrials.gov) were phase 3, randomized, double‐blind, placebo‐controlled clinical trials designed to evaluate the efficacy and safety of lorcaserin in persons with obesity or overweight. Unlike BLOOM and BLOSSOM, BLOOM‐DM was specific for persons with type 2 diabetes mellitus. BLOOM was a 2‐year trial, whereas BLOSSOM and BLOOM‐DM were 1‐year studies.

In all studies, patients received lorcaserin 10 mg BID or matched placebo. BLOSSOM and BLOOM‐DM also included a lorcaserin 10‐mg once daily study arms, but these data are not included, given that lorcaserin 10 mg BID is the FDA approved dose. All patients in these studies were also prescribed diet and exercise. Analyses presented herein evaluated weight loss of lorcaserin (10 mg BID) versus placebo in persons with obesity or overweight persons with OSA from pooled data from the BLOOM, BLOSSOM and BLOOM‐DM trials collected through week 52. Persons with a history of OSA were identified only from patient medical history. Patients did not undergo a sleep study as part of the weight loss study.

### End points and assessments

Primary end points for these analyses follow the key efficacy end point in the pivotal studies, including proportion of persons with a reduction in the baseline body weight of 5% or more at the end of year 1, change in weight between baseline and the end of year 1 and proportion of persons with a reduction in the baseline body weight of 10% or more at the end of year 1. Additional end points analysed included change in heart rate between baseline and the end of year 1 and changes in systolic as well as diastolic blood pressure between baseline and the end of year 1. An analysis looking at blood pressure and heart rate for patients who responded to therapy was also conducted. Safety was not evaluated for the OSA subgroup separately in this analysis, as the safety results were not expected to be different than the overall population in BLOOM, BLOSSOM and BLOOM‐DM 25, 26, 27.

Statistical analyses for all end points in this manuscript were carried out with methods consistent with those published previously for the three pivotal trials 25, 26, 27. The population for this analysis was focused on those patients who had OSA, defined as noted earlier. The modified intention‐to‐treat (MITT) patient group was used for all assessments. Statistical comparisons of lorcaserin versus placebo were performed for the MITT population at week 52 (1 year) with last observation carried forward for missing data.

## Results

### Participants

In the overall pooled data from BLOOM, BLOSSOM and BLOOM‐DM (*N* = 6,636; patients enrolled into lorcaserin 10 mg BID and placebo arms who had baseline and post‐baseline data), there were a total of 336 patients (5%) with OSA in the MITT population. Among persons with OSA, demographics and baseline characteristics (including age, gender, race, BMI and body weight) were similar in the lorcaserin and placebo treatment groups (Table 1). Persons with OSA also had similar demographic and baseline characteristics as patients who did not have OSA (Table 1). However, the OSA group tended to be heavier, taller, older and male. The higher percentage of male patients may explain the differences in height and weight, despite the BMIs appearing similar between the two groups.

**Table 1:** Baseline clinical and demographic characteristics.

	OSA		No OSA	
	Lorcaserin (n = 176)	Placebo (n = 160)	Lorcaserin (n = 3,174)	Placebo (n = 3,128)
Gender, *n* (%)				
Female	78 (44.3)	59 (36.9)	2,591 (81.6)	2,539 (81.2)
Male	98 (55.7)	101 (63.1)	583 (18.4)	589 (18.8)
Age (year)				
Mean (SD)	51.3 (8.68)	51.5 (9.51)	44.3 (11.57)	44.5 (11.45)
Median	51.0	53.5	45.0	45.0
Range	24–65	19–65	18–66	18–66
CV (%)	16.9	18.5	26.1	25.7
Age group, *n* (%)				
18–24	1 (0.6)	1 (0.6)	169 (5.3)	154 (4.9)
25–34	7 (0.4)	9 (5.6)	526 (16.6)	520 (16.6)
35–44	30 (17.0)	28 (17.5)	854 (26.9)	843 (27.0)
45–54	67 (38.1)	50 (31.3)	921 (29.0)	911 (29.1)
55–65	72 (40.3)	72 (45.0)	703 (22.1)	699 (22.3)
>65	0	0	1 (<0.1)	1 (<0.1)
Race *n* (%)				
White or Caucasian	136 (77.3)	131 (81.9)	2,124 (66.9)	2,081 (66.5)
Black or African American	26 (14.8)	14 (8.8)	604 (19.0)	597 (19.1)
Asian	3 (1.7)	1 (0.6)	31 (1.0)	25 (0.8)
Native North American	0	2 (1.3)	18 (0.6)	14 (0.4)
Hispanic or Latino	9 (5.1)	11 (6.9)	368 (11.6)	382 (12.2)
Hawaiian/Pacific Islander	1 (0.6)	0	8 (0.3)	9 (0.3)
Other	1 (0.6)	1 (0.6)	21 (0.7)	20 (0.6)
Weight (kg)				
Mean (SD)	109.3 (18.6)	111.5 (16.9)	100.0 (15.5)	99.7 (15.8)
Median	108.5	110.3	98.5	98.0
Range	73.1–152.8	70.9–156.4	62.6–158.0	53.0–165.2
CV (%)	17.1	15.1	15.5	15.9
Height (cm)				
Mean (SD)	172.6 (9.2)	172.5 (10.0)	166.4 (8.6)	166.4 (8.5)
Median	172.7	172.8	165.1	165.1
Range	148.0–193.0	149.9–203.2	138.2–198.0	139.5–198.1
CV (%)	5.3	5.8	5.2	5.1
BMI (kg m^−2^)				
Mean (SD)	36.5 (4.4)	37.4 (4.1)	36.0 (4.3)	35.9 (4.2)
Median	36.5	37.5	35.6	35.4
Range	26.8–45.7	26.7–45.0	26.8–53.0	26.8–46.5
CV (%)	11.9	11.1	11.8	11.7
BMI group (kg m^−2^)				
≤30	11 (6.3%)	3 (1.9%)	177 (5.6%)	148 (4.7%)
>30 but ≤35	56 (31.8%)	45 (28.1%)	1,263 (39.8%)	1,326 (42.4%)
>35 but ≤40	69 (39.2%)	70 (43.8%)	1,086 (34.2%)	1,037 (33.2%)
>40 but ≤45	38 (21.6%)	42 (26.3%)	636 (20.0%)	602 (19.2%)
>45	2 (1.1%)	0	0	15 (0.5%)

BMI, body mass index; CV, cardiovascular; OSA, obstructive sleep apnea.

### Weight loss

**Figure 1:**
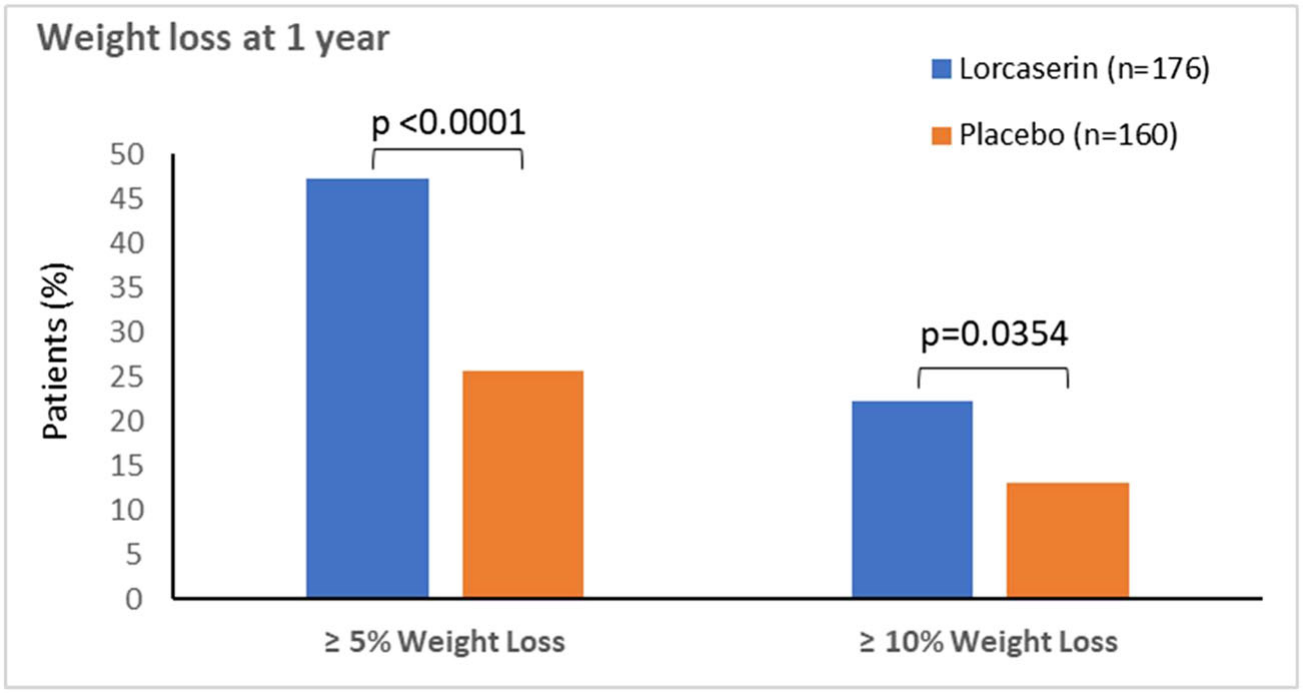
Persons with obstructive sleep apnea who lost ≥5 or 10% of body weight.

**Figure 2:**
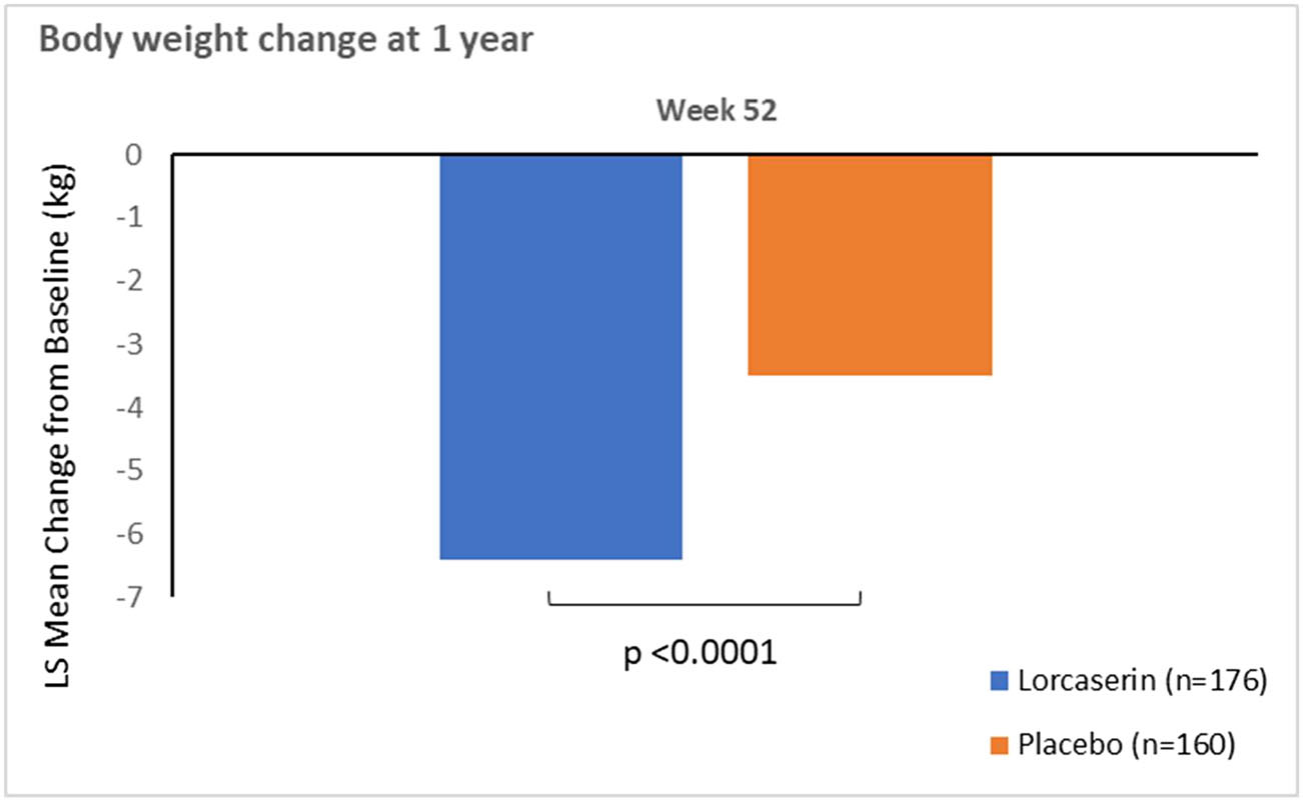
Body weight change in persons with obstructive sleep apnea. LS, least squares.

The proportions of persons with OSA at baseline who achieved ≥5% and ≥10% weight loss at 1 year are shown in Figures 1 and 2. Results for body weight mean change from baseline at 1 year are summarized in Table 2. At the end of year 1, 47.2% of patients receiving lorcaserin had lost 5% or more of their baseline body weight, as compared with 25.6% of patients receiving placebo (*p* < 0.0001) (Figure 1). More patients lost 10% or more of their baseline body weight in the lorcaserin group (22.2%) than in the placebo group (13.1%, *p* < 0.0354) (Figure 1). Patients in the lorcaserin group lost an average of 6.4 kg of the baseline body weight at 1 year, as compared with 3.5 kg in the placebo group (*p* < 0.0001) (Figure 2). Weight loss for persons with OSA was similar as persons without OSA across the three individual studies (Table 3).

**Table 2:** Body weight change for persons with OSA persons at year 1 (MITT population).

	Lorcaserin (n = 176)	Placebo (n = 160)
Body weight		
Baseline, kg (SD)	109.5 (18.5)	111.4 (16.8)
Mean change, kg (SD)	−6.3 (0.5)	−3.5 (0.5)
LS mean change, % (SD)	−6.4 (0.5)*	−3.5 (0.5)

LS, least squares; MITT, modified intention‐to‐treat; OSA, obstructive sleep apnea.

* *p* < 0.0001.

**Table 3:** Weight changes at year 1 in persons with and without OSA from individual studies (MITT population).

	BLOOM		BLOSSOM		BLOOM‐DM	
	OSA (n = 126)	No OSA (n = 2,911)	OSA (n = 142)	No OSA (n = 2,960)	OSA (n = 68)	No OSA (n = 431)
Lorcaserin	−7.0 (0.8)	−5.7 (0.2)	−6.9 (0.8)	−5.7 (0.2)	−3.9 (0.9)	−5.1 (0.3)
Placebo	−3.1 (1.0)	−2.1 (0.2)	−4.2 (0.8)	−2.8 (0.2)	−2.5 (0.9)	−1.8 (0.3)

LS mean changes in weight from baseline at 1 year in individual BLOOM, BLOSSOM and BLOOM‐DM studies for persons with and without OSA (MITT population). LS, least squares; MITT, modified intention‐to‐treat; OSA, obstructive sleep apnea.

### Blood pressure and heart rate

**Figure 3:**
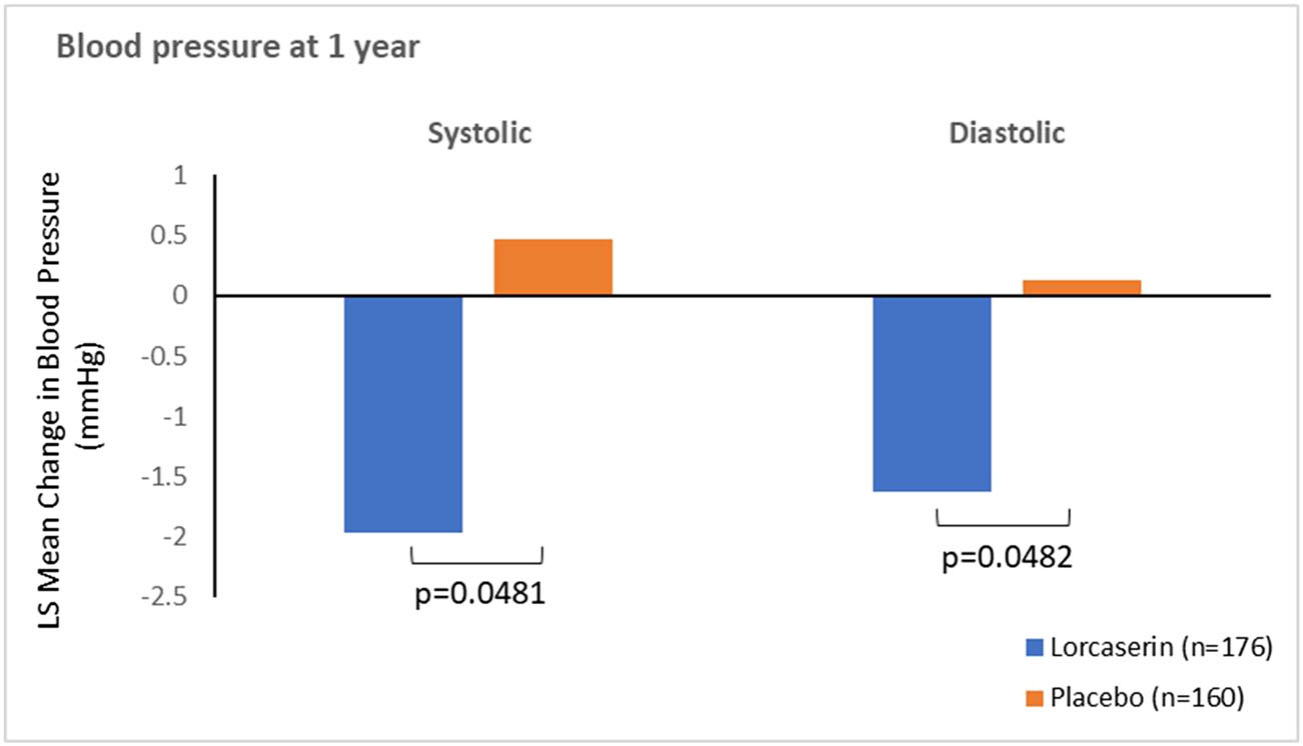
Change in blood pressure in persons with obstructive sleep apnea. LS, least squares.

The results from the change from baseline to 1 year in systolic and diastolic blood pressure analysis and heart rate in persons with OSA at baseline are summarized in Figure 3 and Table 4. At the end of year 1, the mean systolic blood pressure had decreased from baseline by 2.0 mmHg in patients receiving lorcaserin compared with an increase of 0.5 mmHg for patients receiving placebo (Figure 3). The least squares (LS) mean difference between lorcaserin and placebo was statistically significant (difference in LS means, 95% CI: −2.43, 4.84, −0.02; *p* = 0.0481). Systolic blood pressure reductions were greater in patients who responded to treatment (i.e. those patients who achieved ≥5% [lorcaserin: −4.5 mmHg; placebo: −2.4 mmHg] or ≥10% [lorcaserin: −6.3 mmHg; placebo: −0.3 mmHg] weight loss at 1 year).

**Table 4:** Heart rate change from baseline at 1 year in persons with OSA (MITT population).

	Lorcaserin (n = 176)	Placebo (n = 160)
Heart rate, beats per min		
LS mean (SE)	−1.9 (0.6)‡	−0.1 (0.6)

Heart rate change from baseline at 1 year in persons with OSA from pooled data from BLOOM, BLOSSOM and BLOOM‐DM studies (MITT population; LS, least squares; MITT, modified intention‐to‐treat; OSA, obstructive sleep apnea).

Likewise, at the end of year 1, the mean diastolic blood pressure had decreased from baseline by −1.6 mmHg in patients receiving lorcaserin compared with an increase of 0.1 mmHg for patients receiving placebo (Figure 3). The LS mean difference between lorcaserin and placebo was statistically significant (difference in LS means, 95% CI: −1.77, −3.52, −0.01; *p* = 0.048). Diastolic blood pressure reductions were also greater in patients who achieved ≥5% (lorcaserin: −4.8 mmHg; placebo: −4.1 mmHg) or ≥10% (lorcaserin: −4.1 mmHg; placebo: −2.6 mmHg) weight loss at 1 year.

At the end of year 1, the heart rate pressure had decreased from baseline by 1.9 beats per minute (bpm) in patients receiving lorcaserin compared with an increase of 0.1 bpm for patients receiving placebo (Table 4). The LS mean difference between lorcaserin and placebo was statistically significant (difference in LS means, 95% CI: −1.8, −3.57, −0.8; *p* = 0.04). Similar to the blood pressure results, heart rate pressure reductions were greater in patients who achieved ≥5% (lorcaserin: −3.4 bpm; placebo: −2.2 bpm) or ≥10% (lorcaserin: −4.7 bpm; placebo: −3.5 bpm) weight loss at 1 year.

## Discussion

Our analysis finds that persons with OSA treated with lorcaserin experienced significant weight loss relative to patients receiving placebo. Importantly, persons with OSA lost just as much weight as those without OSA, as results were consistent with the weight loss results from the overall population from the BLOOM, BLOSSOM and BLOOM‐DM studies. These results may be more significant considering report of recently reviewed evidence suggesting that OSA leads to weight gain by causing changes that adversely impact the balance intake and expenditure of energy 28. A weight loss of 5–10% is meaningful for patients and is associated with beneficial effects in a number of conditions, including sleep apnea, hypertension, diabetes, dyslipidemia and arthritis. In addition, weight loss and lifestyle modifications can also help prevent the development of heart disease and type 2 diabetes 29, 30, 31, 32. Lorcaserin phase III data show that this is achievable in patients living with OSA 25, 26, 27.

Lorcaserin was also associated with significant improvements in blood pressure and heart rate in persons with OSA relative to placebo, and, as expected, reductions were greater in patients who responded to treatment. For example, in this analysis, the difference in mean systolic BP for lorcaserin relative to placebo was 2 mmHg in the OSA population, with larger reductions in the subgroup of persons with OSA who responded to treatment. These differences could be clinically meaningful as guidelines have noted significant decreases in CV mortality (by 4–5% for coronary heart disease and 6–8% for stroke) are associated in systolic blood pressure reductions of just 2 to 3 mmHg 33.

There are several limitations of this analysis. First, the analysis has been conducted retrospectively and were not prospectively defined. OSA was not objectively measured (i.e. patients self‐identified in medical history) and so there is the potential that it is underreported, as it is considered to be in the community at large. In addition, persons with OSA were not asked compliance with treatment as it is possible that patients who are compliant could have better weight loss than patients that are not compliant. Given the low rates of diagnosis of OSA as well as the reliance of patient self‐report of the diagnosis, there is the potential that many more persons with OSA were included in the ‘non‐OSA’ group, and therefore, this may limit the meaning that can be drawn between the two groups in this exploratory, post hoc analysis. Finally, this analysis did not include a measure of OSA reduction (e.g. apnea–hypopnea index reduction), but rather only weight loss and blood pressure and therefore a preliminary assessment of the impact of weight loss on OSA could not be assessed with these data.

The results from this analysis indicate further research is warranted and prospective efforts are already underway. The effect of lorcaserin on OSA will be prospectively evaluated in a phase 3 study evaluating the effect of long‐term treatment with lorcaserin on the incidence of major adverse CV events in persons with obesity and overweight persons with CV disease or multiple CV risk factors 34. In addition to CV outcomes, this study will provide a large data set (3,500 patients) to assess weight loss, cardiometabolic outcomes, quality of life and surrogates for OSA in persons with pre‐diabetes and sleep apnea.

In summary, this retrospective, subgroup analysis in persons with OSA from three randomized, controlled studies showed significant and meaningful weight loss reduction as well as blood pressure and heart rate reduction for lorcaserin relative to patients receiving placebo. In this analysis, persons with OSA lost just as much weight as those that do not have OSA, important given the clear benefit of weight loss for individuals with OSA. Further prospective research is warranted to evaluate impact of weight loss on OSA and overall outcomes for patients.

## Abbreviations

BID: twice daily
bpm: beats per minute
CPAP: continuous positive airway pressure
CV: cardiovascular
LS: least squares
MITT: modified intention‐to‐treat
OSA: obstructive sleep apnea

## Conflict of interest statement

Ken Fujioka has served as a paid consultant for Orexigen, Novo Nordisk, Shire, Eisai, Gelesis, Ambra, Nazura, Zafgen and KVK Tech; has received research funding from EnteroMedics, Orexigen, Novo Nordisk, Shire and Eisai; and has been a speaker for Orexigen, Novo Nordisk and Shire. Carlos Perdomo and Manoj Malhotra are employees of Eisai, Inc.
